# On the PATHGROUPS approach to rapid small phylogeny

**DOI:** 10.1186/1471-2105-12-S1-S4

**Published:** 2011-02-15

**Authors:** Chunfang Zheng, David Sankoff

**Affiliations:** 1 Département d’informatique et de recherche opérationnelle, Université de Montréal, Canada; 2 Department of Mathematics and Statistics, University of Ottawa, Canada

## Abstract

We present a data structure enabling rapid heuristic solution to the ancestral genome reconstruction problem for given phylogenies under genomic rearrangement metrics. The efficiency of the greedy algorithm is due to fast updating of the structure during run time and a simple priority scheme for choosing the next step. Since accuracy deteriorates for sets of highly divergent genomes, we investigate strategies for improving accuracy and expanding the range of data sets where accurate reconstructions can be expected. This includes a more refined priority system, and a two-step look-ahead, as well as iterative local improvements based on a the median version of the problem, incorporating simulated annealing. We apply this to a set of yeast genomes to corroborate a recent gene sequence-based phylogeny.

## Background

Many comparative genomic problems, such as the median [1], quartet [2], small phylogeny [3], halving [4] or aliquoting [5] problems, require the reconstruction of unknown ancestral genomes, more specifically their gene orders, given the orders in one or more contemporary, genomes. At the heart of many reconstruction methods seeking a most economical solution in terms of genomic distance (or rearrangement distance) is the strategy of maximizing the number of cycles in breakpoint graphs [4,6,7]. We recently introduced PATHGROUPS, a data structure that is designed entirely for this type of strategy [2], and implemented it for the quartet problem. PATHGROUPS is a compact and highly cross-referenced way of storing partially completed cycles, so that genome-wide greedy searches are rapidly executed and the data base quickly updated. A key advantage of the method, and what sets it apart from other techniques, is that its worst case running time depends only linearly on genome size and not at all on the rearrangement distances among the input genomes, while the run time of other reconstruction methods are highly dependent on distance, so that they are not feasible for the large instances of biological interest. A trade-off against its efficiency is that as the distances increase, the PATHGROUPS approach becomes less precise.

In this paper, we present the first implementation of PATHGROUPS for the small phylogeny problem, i.e., for a given unrooted binary tree with a number of given genomes as leaves, to infer the ancestral genomes so that the total branch length (in terms of genomic distance) of the tree is minimized. The computational complexity of the median problem, which is just the small phylogeny problem with only one ancestor, is NP-hard under rearrangement distances, as reviewed in [8] and [9], and so hence is the small phylogeny problem. Thus it is not unexpected that any efficient method will be imprecise for some instances. Nevertheless, given the importance of the small phylogeny problem for evolutionary genomics, and the paucity of methods able to handle highly rearranged genomes containing many thousands of genes, it is worthwhile to try to improve the accuracy of the PATHGROUPS approach, and to extend the range of instances for which it is precise, without sacrificing its computational efficiency.

We first sketch the notion of genomic distance and formalize the small phylogeny problem. We then present our approach to small phylogeny, including a greedy algorithm which exploits the PATHGROUPS data structure efficiently, making use of a simple, rapidly updated, priority system - basically a one-step look-ahead - to choose the next step. We assess the accuracy of the method and implement various techniques to improve it. The first is a new, more refined, system of priorities for the median problem. The second improvement is a two-step look-ahead for the median. The final experiments improve the full small phylogeny solution by iteratively recalculating each ancestral genome, using the median instance of the method, and incorporating a simulated annealing technique to avoid local optima. We assess the extent to which these techniques improve the results, and their computational costs.

As applied to a number of yeast gene orders, we show that gene order data confirm the phylogeny previously obtained from gene sequence data.

## Methods

### Preliminaries

#### Genomes and rearrangement operations

We model the evolutionary rearrangement of a genome containing *n* distinct signed genes through the accumulated operation of number of processes familiar in classical genetics: inversion, reciprocal translocation, transposition, chromosome fusion and fission, operating on linear chromosomes. We will not delve into the details of the operations; formally they can all be subsumed under a single operation called double-cut-and-join (DCJ) which need not be described here. All that is needed for our purposes is a formula due to Yancopoulos *et al.*[10] that gives *d*(*G*_1_, *G*_2_), the minimum number of rearrangement operations needed to transform one genome into another in terms of properties of the “breakpoint graph” determined by *G*_1_ and *G*_2_, the initial and final genomes. To calculate *D* efficiently, we construct and analyze the breakpoint graph as follows.

For each genome, each gene *g* with a positive polarity is replaced by two vertices representing its two ends, i.e., by a “tail” vertex and a “head” vertex in the order *g_t_*, *g_h_*; for –*g* we would put *g_h_*, *g_t_*. Each pair of successive genes in the gene order defines an adjacency, namely the pair of vertices that are adjacent in the vertex order thus induced. For example, if *i*, *j*, –*k* are three neighbouring genes on a chromosome then the unordered pairs {*i_h_*, *j_t_*} and {*j_h_*, *k_h_*} are the two adjacencies they define.

If there are *m* genes on a chromosome, it has determined 2*m* vertices by this stage. The first and the last of these vertices are called telomeres. We convert all the telomeres in genome *G*_1_ and *G*_2_ into adjacencies with additional vertices all labelled *T*_1_ or *T*_2_, respectively. The breakpoint graph has a blue edge connecting the vertices in each adjacency in *G*_1_ and a red edge for each adjacency in *G*_2_. We make a cycle of any path ending in two *T*_1_ or two *T*_2_ vertices, connecting them by a red or blue edge, respectively, while for a path ending in a *T*_1_ and a *T*_2_, we collapse them to a single vertex denoted “T”.

Each vertex is now incident to exactly one blue and one red edge. This bicoloured graph decomposes uniquely into *κ* alternating cycles. If *n*′ is the number of blue edges, then [10]:

*d*(*G*_1_, *G*_2_) = *n*′ – *κ.*       (1)

**Figure 1:**
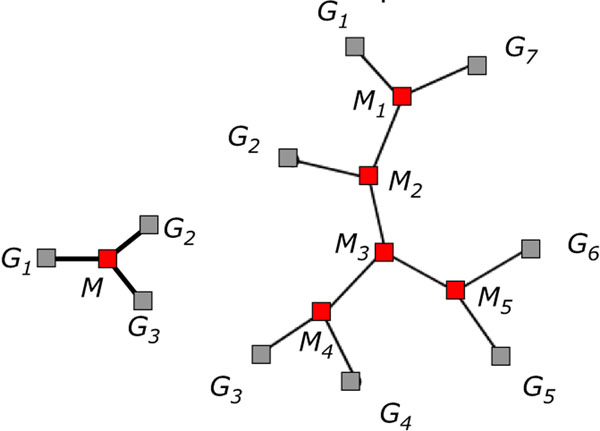
Representation of the median (left) and more general small phylogeny (right) problems. Grey squares indicate given genomes, red squares those to be reconstructed. Each line connecting two genomes represents a breakpoint graph and a distance.

For a given unrooted binary tree *T* on *N* given genomes *G*_1_, *G*_2_, ⋯, *G_N_* (and thus with *N* – 2 unknown ancestral genomes *M*_1_, *M*_2_, ⋯, *M_N_*_–2_ and 2*N* – 3 branches) as depicted in Fig. 1, *the small phylogeny problem is to infer the ancestral genomes so that the total edge length of T*, *namely ∑_XY∈E_*_(_*_T_*_)_*d*(*X*, *Y*), *is minimal.*

The computational complexity of the median problem, which is just the small phylogeny problem with *N =* 1, is known to be NP-hard and hence so is that of the general small phylogeny problem. Our method will be shown to run in linear time, so obviously it is not guaranteed to find an exact solution. One of the goals of this paper is to determine for what range of instances PATHGROUPS leads to accurate solutions, and how the approach may be improved to extend this range.

### Data structure and algorithm

In this section we first discuss PATHGROUPS in some detail as it applies to the median problem with three given genomes and one ancestor to be reconstructed. Then we describe how this works for the simultaneous reconstruction of all the ancestors in the small phylogeny problem.

#### Paths and fragments

We generalize our definition of a path to be any connected subgraph of a breakpoint graph, namely any connected part of a cycle. Initially, each blue edge in the given genomes is a path.

A *fragment* is any set of genes connected by red edges in a linear order. The set of fragments represents the current state of the reconstruction procedure. Initially the set of fragments contains all the genes, but no red edges, so each gene is a fragment by itself.

#### Pathgroups

The objective function for the small phylogeny problem consists of the sum of a number of genomic distances, one distance for every branch in the phylogeny. Each of these distances corresponds to a breakpoint graph. A given genome determines blue edges in one breakpoint graph, while the red edges correspond to the ancestral genome being constructed. For each such ancestor, *the red edges are identical in all the breakpoint graphs corresponding to distances to that ancestor.* A pathgroup is a set of three paths, all beginning with the same vertex, one path from each partial breakpoint graph currently being constructed. Initially, there is one pathgroup for each non-*T* vertex. (We do not construct pathgroups for each *T* vertex separately, to be explained later, though paths ending in *T* vertices are found in other pathgroups.)

Pathgroups overlap because most paths are in two pathgroups, one associated with its initial vertex and one with its final vertex. With respect to a given path *xy*, we say the pathgroup determined by vertex *x* is the partner of the pathgroup determined by *y*. For the kind of binary (or bifurcating) trees we use, each pathgroup may have up to three distinct partners.

#### Priorities

Our main algorithm aims to construct three breakpoint graphs with a maximum aggregate number of cycles. At each step it adds an identical red edge to each path in the pathgroup, altering all three breakpoint graphs. This removes two (partner) pathgroups, turning one or more of their paths into cycles and concatenating each of their remaining paths with a path in some other pathgroup. We do not add red edges incident to *T* vertices. It is always possible to create one cycle, at least, (if not all the paths in the pathgroup end in *T*) by adding a red edge between the two ends of any one of the paths. The strategy is to create as many cycles as possible. If alternate choices of steps create the same number of cycles, we choose one that sets up the best configuration for the next step. Thus the pathgroups are prioritized,

1. first by the maximum number of cycles that can be created within the group, without giving rise to circular chromosomes.

2. second, for those pathgroups allowing equal numbers of cycles, by considering the maximum number of cycles that could be created in the next iteration of step 1, in any one pathgroup affected by the current choice.

A pathgroup may receive no priority, if creating any cycle within the pathgroup necessarily creates a circular chromosome. Note that in adding a red edge *xy*, this causes not only the disappearance of two partnered pathgroups, but it also changes paths in other pathgroups, which we call secondary pathgroups. Furthermore, each *secondary* pathgroup may itself have partner pathgroups whose paths, though not affected by the addition of *xy*, may have changed priorities. We call these *tertiary* pathgroups.

**Figure 2:**
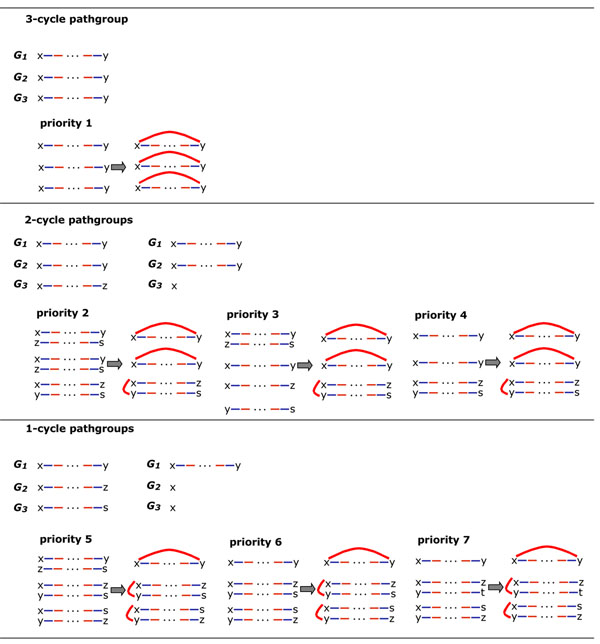
Priorities of all pathgroups of form [(*x*, *a*), (*x*, *b*), (*x*, *c*)] for inserting red edges, for each ancestral vertex in the median problem. Includes sketch of three paths in “*x*” pathgroup plus other paths involved in calculating priority. For example, completing the pathgroup [(*x*, *y*), (*x*, *y*), (*x*, *z*)] by adding the red edge *xy* always produces two cycles, but can set up a pathgroup with 3 potential cycles (priority 2), 2 potential cycles (priority 3) or 1 potential cycles (priority 4).

The pathgroups and the priorities are illustrated in Figure 2.

#### The *makeCycles* algorithm

By maintaining a list of pathgroups for each priority level, and a list of fragment endpoint pairs (initial and final), together with appropriate pointers, the algorithm **makeCycles** requires *O*(*n*) running time.

Algorithm makeCycles

**input:** pathgroups each consisting of three blue one-edge paths

**output:** ancestral genome

**while:** there remain pathgroups with priorities

1. add red edge to pathgroup of highest priority, creating at least one cycle, thus deleting this pathgroup and its partner.

2. update the paths in the secondary pathgroups affected by the addition of the red edge, and update the red fragment extended by this edge or created by the joining together of two existing red fragments.

3. update the priorities of the secondary pathgroups, the tertiary pathgroups, and the at most two pathgroups associated with the endpoints of the red fragment extended or created in step 2.

Not all the red fragments output by **makeCycles** are complete chromosomes of the ancestral genome; they may just be chromosome fragments. We know

**Proposition [2]:***Adding a red edge xy in a pathgroup creates at most four secondary pathgroups and atmost eight tertiary pathgroups*.

The proposition, together with the two facts:

1. the total number of pathgroups decreases by two by each step

2. the calculation or recalculation of the priority of each pathgroup requires constant time

ensure the *O*(*n*) running time of the algorithm. If we had allowed red edges to connect *T* vertices or allowed pathgroups determined by *T* vertices, the number of potential secondary and tertiary pathgroups affected by the addition of a red edge would have increased considerably, depending on the number of chromosomes in the genomes, but this would not have affected the *O*(*n*) property.

Seven priority levels are illustrated in Figure 2, based on the construction of

1. three cycles,

2. two cycles setting up a) three, b) two or c) one in the look-ahead, or

3. one cycle setting up a) three, b) two or c) one in the look-ahead.

Note that when a red edge is defined, the pathgroup is emptied, either by the creation of cycles, or by the integration of *x* as a non-endpoint of some path.

An example of the solution to the median problem is given as Additional File 1.

#### Small phylogeny

To apply PATHGROUPS to the small phylogeny problem, we set up an entire set of pathgroups for every internal (ancestral) node. Initially, in the pathgroups for those ancestral nodes connected to two given genomes, one of the paths will be missing and replaced by a single vertex of the breakpoint graph, as illustrated in Figure 2. Those ancestral nodes connected to only one given genome will have two missing paths in each pathgroup, both replaced by the vertex. Finally those ancestral nodes connected only to other internal nodes will have all paths missing in each pathgroup, all replaced by the vertex.

As the algorithm executes, a pathgroup with the highest priority found among any of the internal nodes is chosen to be processed next. Pathgroups connected to two given nodes will tend to be processed first, building up all three paths and combining the pathgroups one by one. *Each time a red edge is added to a path*, *this becomes a blue edge in the corresponding pathgroup for ancestral genome*(*s*) *connected to it.*

Eventually even the nodes furthest from any given genomes will accumulate enough edges in their pathgroups so that cycles can be formed and so that fragments of the associated genomes begin their reconstruction.

## Results and discussion

We implemented **makeCycles**, adapted for the small phylogeny problem, so that it could achieve its worst case linear run time capability.

### Improving accuracy

As mentioned in the Introduction, we do not expect to guarantee exact solutions for NP-hard problems using a linear time algorithm. Moreover, because this is a single-pass method, we cannot even expect to find locally optimal solutions. Thus we must investigate how close is the approximation and what are the prospects for improvement.

We undertook a series of simulations of the method and of its improvements as described below. All genomes had length *n =* 5000. Each data point represents the average of ten simulations. Trees were generated from a ten-chromosome ancestor by 90 % chromosomal inversions at randomly and independently chosen breakpoints, and 10 % reciprocal translocations. The simulations were performed on a MacBook Pro with 3.06 GHz processor speed.

**Figure 3:**
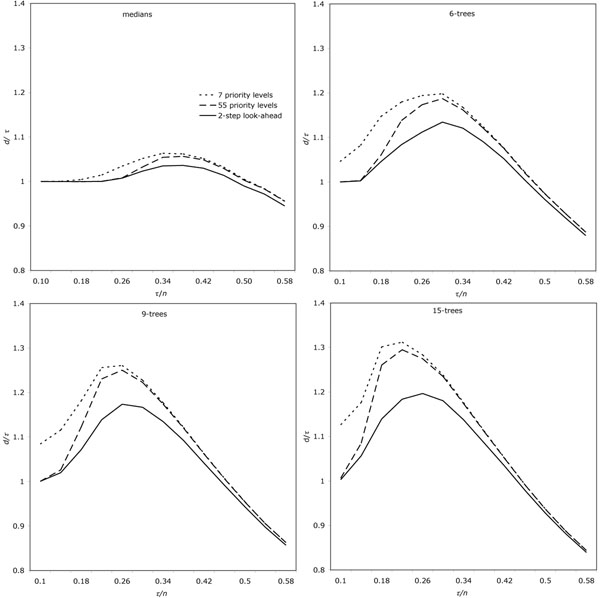
Effect of refined priority and two-step look-ahead on error. *τ =* total branch length in tree with known simulated ancestors, *n =* number of genes per genome, *d* = total branch length in tree with reconstructed ancestors. Averages of 10 runs.

In Figure 3, we assess the accuracy of the method by comparing, on the vertical axis, *d*, the total distance inferred by our method, with *τ*, the true number of rearrangements inherent in the simulated tree, as measured along all branches, whether they connect given or simulated genomes. The notation *τ* is used slightly differently on the horizontal axis, where it represents the number of rearrangements actually performed on each branch of the tree. The figure depicts (dotted lines) how the results of our method, which is accurate (i.e., *d/τ ≈* 1) for 3-trees (medians) and for rearrangement rates up to about 0.2*n*, but increasingly inaccurate for larger trees.

Note that the large decrease in *d/τ*, starting at *τ/n* in the range of 0.35 to 0.4, is illusory. Since *d* for any one branch cannot exceed *n*, a well-known property of rearrangement distance, while we can increase *τ* indefinitely, inference algorithms inevitably find shorter derivations of ancestral genomes than were actually used in generating them. Thus the entire right hand area of the figures, reproduced here for completeness, is of little interest.

#### Refining the priorities

As a first improvement, we refine the priority levels as follows. We retain the number of cycles created as the primary classification, so that for example a two-cycle pathgroup always has higher priority than a one-cycle pathgroup. And we continue to refine this classification by taking account of the best new pathgroup that would be set up by processing the pathgroup under examination. In addition, however, to provide another level of refinement, we check *all* the pathgroups that would be affected by processing the pathgroup under examination and count the net change, positive or negative, in potential cycles among all these. This leads to 55 priority levels. As is seen in Figure 3, this refinement has a dramatic effect both in decreasing *d/τ* and in postponing the value of *τ/n* where *d* begins to rise much faster than *τ.*

#### Two-step look-ahead

As a further refinement, we considered the configuration that would be produced two algorithmic steps beyond the current step. Here, after identifying which one or several of the potential (after one step) pathgroups could produce the largest number of cycles by the addition of a red edge, we check what would happen after processing such a pathgroup, namely of the new (second step) pathgroups created, what is the largest number of cycles (1, 2 or 3) any one of them could produce by adding a red edge. This sub-classifies the 55 priorities three ways, creating a system with 165 priority levels.

As is clear in Figure 3, this additional step improves the accuracy even more than the first refinement did. The effect on reducing *d/τ* is especially strong, while there is little additional effect in delaying the point at which *d* begins to rise more quickly than *τ.*

The increase in run time caused by the two-step look-ahead is substantial, as we will learn from the next experiment. However, given that these results are based on rather highly arranged genomes containing 5000 genes, and a moderately large phylogeny (15-trees), the cost is hardly prohibitive.

#### Iterative local improvement based on the median

Searching for improvements in ancestral genome reconstruction by iteratively applying the median version at each ancestral node, accepting the changes only if they lower the objective function, is a time-honoured strategy in phylogenetics, including in rearrangement phylogeny [3,11]. This would seem particularly appropriate in the present context since the median version of pathgroups is rapid and, as we have seen, relatively accurate.

Preliminary trials indicated that this procedure would be susceptible to premature capture by local minima. Experimenting with various regimes of simulated annealing, we settled on simply accepting every median reconstruction that was better than *or equal* to the existing reconstruction, and stopping after a predetermined number of steps.

**Figure 4:**
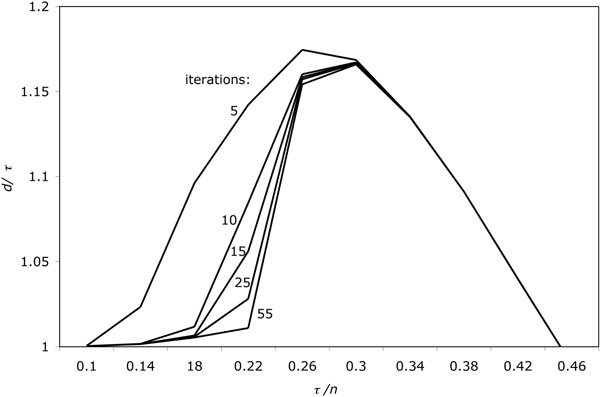
Iterative improvement of solution to small phylogeny as a function of rearrangement rate. The median reconstruction is based on the two-step look-ahead. Each data point represents the average of 10 runs.

Figure 4 depicts the results of this experiment (on 10-trees) up to 50 iterations. It can be seen that there is again a dramatic improvement in accuracy, both in reducing *d/τ* but also in delaying the point at which *d* begins to rise more quickly than *τ.* Simulations up to 100 iterations showed very small increases in accuracy over these results.

**Figure 5:**
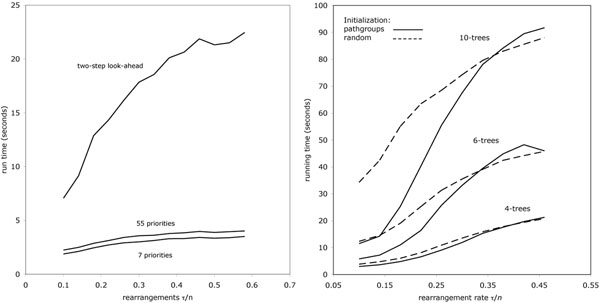
(left)Increase in run time due to refined priority and look-ahead. Based on 15-trees. (right) Dependence of average time for first five iterations on number of leaves in phylogeny and rearrangement rate on branches.

A time analysis (Figure 5) show that the iterative procedure exacts a higher cost than the other refinements. Run times for 50 iterations are ten times those shown, but again not enough to impede regular use of this method.

To what extent is the improvement seen here due to the initialization of the tree using PATHGROUPS and to what extent is it due to the iterative use of the median (also making use of PATHGROUPS of course)? After the necessary 50 or 100 iterations, the process no longer “remembers” its initialization, and neither approach seems more susceptible to falling into local optima. What about computing time? Figure 5 also shows what happens when the reconstructions are initialized with random genomes. With less rearranged genomes, there is a distinct time saving with the PATHGROUPS initialization, especially with larger phylogenies, but this disappears with more highly rearranged genomes.

### Comparison to exact algorithm

The best current method for solving the gene order median problem is ASMedian_linear [12]. This relies on the detection of an “adequate subgraph” in the breakpoint graph, which allows the decomposition of the problem into easier instances. When this method finds adequate subgraphs, it is very efficient; otherwise execution time may be prohibitive. The latter case tends to occur for instances of the median problem where the input genomes are highly rearranged with respect to each other.

For median problems where *τ/n* is less than about 0.3, we have shown that the PATHGROUPS approach, with all its improvements, rapidly produces median solutions are that within a few tenths of one percent of optimal. But as *τ/n* gets larger than 0.3, the solutions become less precise, although execution time remains small.

We compared PATHGROUPS with ASMedian_linear for *n =* 5000 and *τ =* 500,1000 and 1500 random rearrangements on each of the three branches leading from a given median. For twenty runs at each condition, the results were,

• for *τ* = 500, the PATHGROUPS run took about 250 milliseconds each, except one which took around 500, while the ASMedian_linear generally ran in 375 milliseconds, except for two runs requiring several seconds, and one run that did not terminate after 10 minutes. The PATHGROUPS solutions were on the average two tenths of one percent worse than the optimal solutions found by ASMedian_linear.

• for *τ* = 1000, the PATHGROUPS run took about 475 milliseconds each, but only seven of the ASMedian_linear runs terminated in less than a second, and five of them did not terminate, even after an hour. Again the PATHGROUPS solutions were only a few tenths of one percent worse than the optimal solutions found by ASMedian_linear.

• for *τ* = 1500, the PATHGROUPS run took about 920 milliseconds each, but none of the ASMedian_linear runs terminated in less than an hour. The PATHGROUPS solutions were generally just as good as the best solutions found to date by ASMedian_linear after an hour of searching.

In sum, in trading off precision against efficiency, PATHGROUPS sacrifices very little accuracy for genomes that are not highly rearranged, but also continues to give good results for median problems which are too highly rearranged to be solved exactly. Recall that for the small phylogeny problem, the median algorithm is called iteratively, so that execution times of several minutes or hours disqualifies the exact method.

### Yeast small phylogeny including both rapidly and slowly evolving genomes

**Figure 6:**
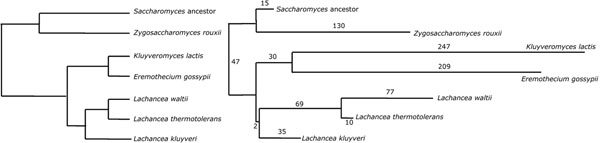
Phylogeny of seven yeasts [14], including pre-WGD *Saccharaomyces ancestor*, from gene sequences (left) and gene order (right).

*Saccharomyces cerevisiae* and its closest relatives are descendants of a whole genome duplication (WGD) event more than 100 million years ago [13]. In a maximum likelihood phylogeny of 79 yeast species inferred from eight gene sequences [14], there are six relatives of *S. cerevisiae* whose genomes have been sequenced, but that diverged before the WGD. These six, plus the manually reconstructed ancestral genome [13] that underwent WGD are depicted on the left of Figure 6. Since their divergence these genomes have evolved at very different rates, with *E. gossypii*, for example, showing a substitution rate over three times as great as *L. kluyveri*, making phylogenetic inference prone to various biases.

We extracted gene orders involving the 4011 sets of orthologous genes these genomes all have in common from the Yeast Gene Order Browser [13]. Gene order rearrangement distances between them shows that the evolution rate for gene order varies in much the same way as for gene sequence, with *E. gossypii*, for example, changing gene order much more rapidly than *L. kluyveri*.

We used PATHGROUPS and the associated algorithm to show that maximum likelihood phylogeny in [14] is also the optimal gene order phylogeny, as depicted on the right of Figure 6, where branch lengths are drawn proportional to the values indicated as inferred by the algorithm. Despite the small branch length defining the *Lachancea* clade, all other phylogenies have excess cost of at least 5, including those where *L. kluyveri* branches from the *K. lactis - E. gossypii* grouping or before the divergence of the latter from the other two *Lachancia* species.

## Conclusions

For genomes that are moderately rearranged, PATHGROUPS an extremely rapid and rather accurate reconstruction of the ancestral genomes in the small phylogeny problem. This is especially true of the two-step look-ahead version of the algorithm. With a small loss of precision, it can rapidly handle instances of the median problem where an exact algorithm may take hours. Thus it can be integrated into a small phylogeny search where the exact algorithm cannot.

We have not investigated efficient memory handling procedures, and this will be required to analyze large phylogenies, since every gene requires two pathgroups for every tree node, and our present implementation associates memory in an unnecessarily profligate way to each of these pathgroups, as well as to the chromosome fragments, in order to achieve time efficiencies.

For small phylogeny problems with more highly rearranged genomes, the question arises of how to use PATHGROUPS. For the iterative approach, if enough computing power is available, it suffices to initialize with random genomes, though of course the PATHGROUPS median remains essential. If a single pass is desirable, however, it is clear that the two-step look-ahead greatly increases the accuracy of the approach.

## Availability

The code for using PATHGROUPS for small phylogeny may be downloaded from http://137.122.149.195/Softwares/

## Competing interests

The authors declare they have no competing interests.

## Supplementary Material

Additional File 1 Solution of a median problem by PATHGROUPS Click here for file
