# Supplementary figures and images for: On the PATHGROUPS approach to rapid small phylogeny
Source: BMC Bioinformatics. 2011 Feb 15;12(Suppl 1):S4. doi: 10.1186/1471-2105-12-S1-S4 (PMC3044296; doi:10.1186/1471-2105-12-S1-S4)

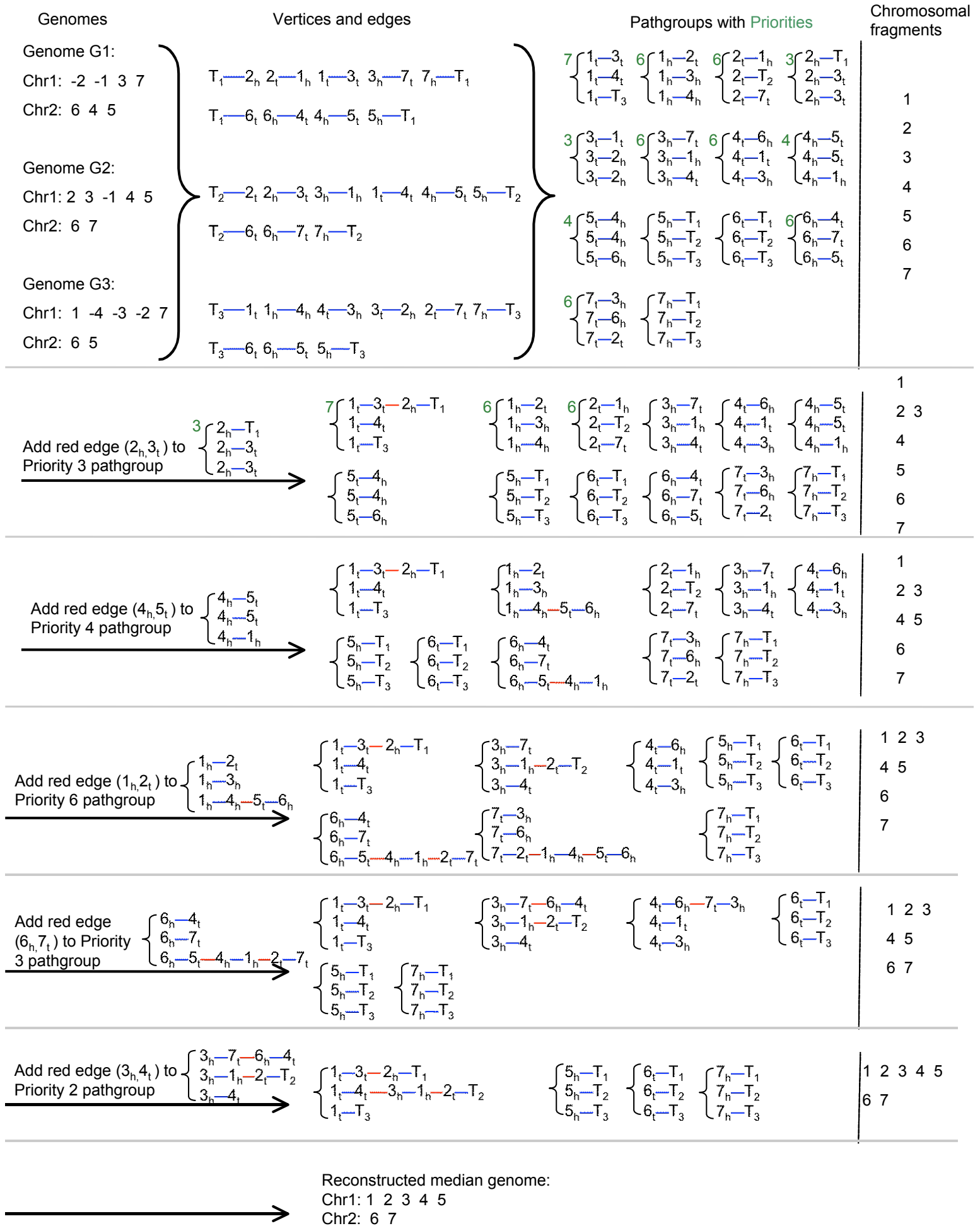

Supplement: Additional File 1 — Solution of a median problem by PATHGROUPS [file 1471-2105-12-S1-S4-S1.pdf]
